# Gottheil

**Published:** 1912-02

**Authors:** 


GOTTHEIL in a review of the progress of Syphilography (*Progressive Medicine,* Vol. XIII, No. 3) deplores the excessive advertising which 606 has received in the lay press. In summing up his experience with the drug he states: "Its immediate effects may be better, in some cases, than that of mercury; in others, it is slower and less certain; in some cases, it fails entirely. It cannot, therefore, replace the mercury-iodine treatment, but should be used in conjunction with it in suitable cases.

In regard to the Wassermann reaction. "While a positive reaction means an infection with syphilis in 99 per cent. of the cases, it cannot be regarded as conclusive without knowing the character of the work done by the serologist. The test is so delicate, and its technic must be so rigorously carried out that one examination should not be relied upon in doubtful cases."

"The test can never supplant competent and exhaustive clinical observation, but can aid it greatly."

Reports of the Wassermann reaction in congenital idiots are positive in 9—14.7 per cent. With this fact before him be advocates that the reaction be tested in every pregnancy and if positive that the mother receive appropriate treatment. Mercurial treatment should also be administered to the mother if there is a specific history regardless of the reaction.

